# Access-Selection Algorithm for Heterogeneous Wireless Networks Based on Uncertain Network Attribute Values

**DOI:** 10.1155/2022/4646889

**Published:** 2022-02-24

**Authors:** Xiaoxue Guo, Mohd. Hasbullah Omar, Khuzairi Mohd Zaini, Jianfeng Gong, Jingcheng Fang, Gen Liang

**Affiliations:** ^1^School of Science, Guangdong University of Petrochemical Technology, Maoming 525000, China; ^2^InterNetWorks Research Laboratory, School of Computing, Universiti Utara Malaysia, UUM Sintok 06010, Kedah, Malaysia; ^3^Department of Computer Engineering, Maoming Polytechnic, Maoming 525000, China; ^4^College of Computer Science and Software Engineering, Shenzhen University, Shenzhen 518060, China; ^5^College of Electronics and Information Engineering, Guangdong University of Petrochemical Technology, Maoming 525000, China

## Abstract

A heterogeneous wireless network (HWN) environment contains many kinds of wireless networks, such as UMTS, LTE, and WLAN, where users move around within their coverage area. How to ensure mobile users select the most suitable network is a hot research topic for HWNs. Owing to the mobility of users, the interference of wireless signals, and the fluctuation of network status, the network attribute values obtained by mobile users are often uncertain. However, the traditional access-selection algorithms assume that mobile users can obtain accurate network attribute values, which makes users unable to access the most appropriate network. To solve this problem, this paper designs an access-selection algorithm for HWNs in the context of inaccurate network attribute values. First, the algorithm calculates the network attribute values based on the hesitant fuzzy theory, then calculates the weights of network attributes using the fuzzy analytic hierarchy process (FAHP), and finally sorts the candidate networks using the hesitant fuzzy technique for order preference by similarity to ideal solution (TOPSIS) method. The simulation results show that the proposed algorithm enables users to select the most suitable network to access under the inaccurate network attribute environment and obtain higher gains.

## 1. Introduction

With the continuous development of wireless communication technology, various wireless communication networks (e.g., mobile cellular networks, wireless local area networks (WLANs), wireless metropolitan area networks (WMANs), and satellite communication networks) are providing users with wireless network services. These wireless networks with different architectures, such as mobile cellular networks, WLANs, and WMANs, constitute heterogeneous wireless networks (HWNs) with overlapping coverage areas [1, 2].

An HWN environment incorporates many kinds of radio access technologies, which have different network parameters and characteristics (including bandwidth, delay, packet loss ratio, and cost). When mobile users perceive that another wireless network can provide better service than the currently connected wireless network, they will switch from one wireless network access point to another. How to make mobile users choose the most suitable network among multiple candidate networks has become one of the research topics in HWNs [3, 4].

The access selection has three main steps with specific functions [5]: first, find a network by collecting data (network performance, service, and user terminal) that can be searched at the location of the mobile user terminal. The collected data will be considered when making a network-selection judgment. Second, make a decision on access to decide which candidate network to select and when to switch according to the access-selection algorithm. Third, enable access by performing access operations according to the calculated result of the second step and the relevant network protocol.

Traditional access-selection algorithms usually adopt mathematical models, such as multiattribute decision making (MADM) [6, 7], utility theory [8, 9], fuzzy logic [10, 11], game theory [12–14], optimal calculation [15, 16], and neural network [17–19]. All these models calculate the scores of candidate networks according to the accurate network attribute values [20]. Owing to the mobility of users, the interference of wireless signals, and the fluctuation of network state, the network attribute values collected by users are often not accurate. Therefore, how to design an access-selection algorithm for HWNs with uncertain network attribute values drives the research proposed herein.

This paper designs a multiattribute access-selection algorithm for HWNs based on uncertain network attribute values, which includes three calculation modules (network attribute value, network attribute weight, and candidate network score). The proposed algorithm first calculates the inaccurate network attribute values based on hesitant fuzzy numbers, then calculates the weights of the network attributes based on the fuzzy analytic hierarchy process (FAHP), and finally calculates the scores of candidate networks using the hesitant fuzzy technique for order preference by similarity to ideal solution (TOPSIS) method to obtain the ranking of candidate network scores, thus enabling users to select the network with the highest score.

The main contributions and features of this paper are as follows:

- This paper provides an access-selection calculation method in the event uncertain network attribute values arise by considering the uncertainty of network attribute values in an HWN environment.
- This paper proposes a hesitant fuzzy TOPSIS method to score candidate networks based on the traditional TOPSIS method.
- This paper designs a network access-selection algorithm which integrates the hesitant fuzzy set theory, the FAHP, and the hesitant fuzzy MADM method. To our best knowledge, a thorough search of the relevant literature yielded zero results related to assigning an access-selection method in an HWN environment with uncertain network attribute values in a similar manner as this paper.
- The proposed algorithm enables users to select the most suitable network, increases the users' gains, and reduces unnecessary handoffs between different networks.

The rest of this paper is organized as follows.  Section 2 reviews the research work related to this article.  Section 3 provides detailed calculation steps of the algorithm. In addition, Section 4 configures simulation environment parameters and discusses the experimental results. Furthermore, Section 5 summarizes the article and introduces further research.

## 2. Related Work

When users run different applications, they have different requirements for network performance and assign different weights of importance to different network attributes [21]. To provide users with a better user experience, the mobile user terminal should select the best network according to the user's service type and wireless network performance in a dynamic HWN environment and switch networks when necessary [22]. To date, a large number of access-selection algorithms have been proposed in related literature.

Habbal et al. [23] put forward a context-aware multiattribute access-selection approach that integrates the context-aware concept and the MADM theory. First, this approach uses AHP to calculate the weight of decision parameters and utilizes the TOPSIS method to select the optimal network. This algorithm can solve the problem of abnormal ranking of candidate networks.

Goyal et al. [24], according to the characteristics of applications in speech, video, and best-effort, first use the utility function to calculate the utility value for these three applications. Then, they use the FAHP based on triangular fuzzy numbers to calculate the weight of attributes. Finally, they use the simple additive weighting (SAW), TOPSIS, and the multiplicative exponential weighting (MEW) to calculate the score of each candidate network.

Ahuja et al. [25] proposed an access-selection algorithm combining the utility function and fuzzy logic. The algorithm uses the utility function to calculate the utility values of received signal strength, available bit rate, signal-to-noise ratio, throughput, and bit error ratio (BER) and utilizes the particle swarm optimization (PSO) to calculate the weights. Finally, the output results are calculated through the fuzzy logic system. This algorithm reduces unnecessary handoffs between networks.

Yu et al. [26] proposed a method to calculate the subjective weights of network attributes and the subjective utility values of candidate networks under four different applications using the FAHP and then obtains the objective weights of network attributes and the objective utility values of candidate networks using the entropy method and the TOPSIS method, respectively. Finally, they select a network whose comprehensive utility value is the highest according to the comprehensive utility value and handoff threshold of each candidate network.

Liang et al. [27] designed an access-selection algorithm combining service characteristics and user preferences. First, the algorithm calculates the utility value of each network attribute for different applications using the utility functions. Then, it adopts the entropy method and the FAHP to calculate the objective weight and subjective weight of network attributes respectively and utilizes the FAHP to calculate the user preference value of applications for the candidate networks. Finally, it uses the MADM method to calculate the score of each candidate network according to the utility values and weights of the network attributes.

Ahuja et al. [28] put forward an access-selection algorithm under the HWN environment composed of UMTS, WLAN, GPRS, and the Worldwide Interoperability for Microwave Access (WiMAX). Considering the different requirements of the voice, video, and data applications, the entropy method is used to calculate the weights of network attributes, which are adjusted according to the requirements of different applications. Finally, the ranking of candidate networks is calculated using the TOPSIS method.

Traditional access-selection algorithms assume that mobile users can obtain accurate network attribute values. However, in heterogeneous wireless network environment, the network attribute values obtained by users are often uncertain. Therefore, in order to enable users to access the most appropriate network in an environment where the network attribute value is uncertain, the algorithm proposed herein integrates the hesitant fuzzy theory, the fuzzy analytic hierarchy process, and the hesitant fuzzy TOPSIS. It calculates the inaccurate network attribute values under different applications based on the hesitant fuzzy theory and calculates the weights of network attributes using the FAHP. Finally, it calculates the ranking of candidate networks with the hesitant fuzzy TOPSIS.

## 3. System Model

**Figure 1:**
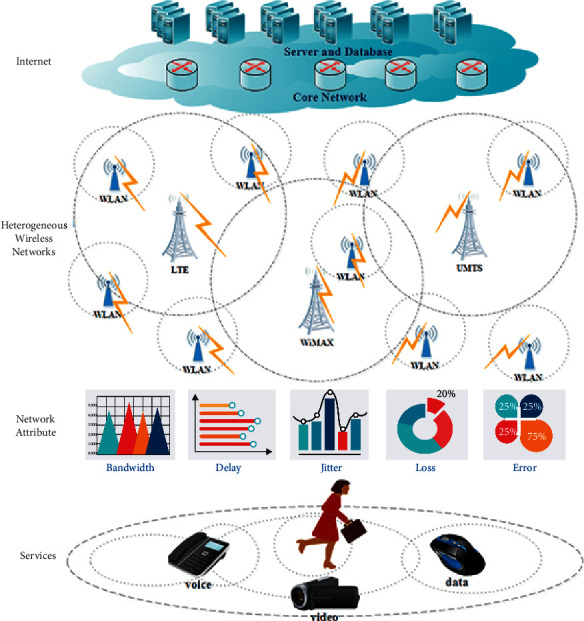
Scenario for heterogeneous wireless network (HWN) access selection.

For the HWNs studied in this paper, there are four kinds of wireless networks: UMTS, LTE, WLAN, and WiMAX, whose coverage areas overlap and coincide with one another. Users can move within the coverage areas and receive the attribute values of each candidate network when they move. In this paper, the network attributes received by users are assumed to include bandwidth, delay, jitter, packet loss ratio, and BER. In addition, this paper assumes that the applications run by users are the voice, video, and data applications. The research scenario of this paper is presented as below (Figure 1).

### 3.1. Calculation of Network Attributes Based on Hesitant Numbers

When users are in an HWN environment, they may receive multiple sets of different network attribute values due to the mobility of the users, the interference of wireless signals, and the fluctuation of wireless network performance. As these network attribute values may be different and users will think that these set of values are all possible, they cannot select a set of values for making a decision on access selection, so users hesitate in the decision-making process of access selection. According to the concept of the hesitant fuzzy set [29, 30], each element of the hesitant fuzzy set is represented by several possible numerical values, which is in-line with the access selection of the HWNs used in this paper. Therefore, this paper calculates the network attributes based on the hesitant fuzzy set. The specific calculation steps are as follows:

**Step 1 .**

Collection and definition of network attribute values based on hesitant fuzzy numbers.

The basic element of the hesitant fuzzy set is the hesitant fuzzy number (also known as the hesitant fuzzy element), and each hesitant fuzzy number contains some possible values. For the convenience of explanation, the hesitant fuzzy set is first defined.

**Definition 1 .**

Let *T* be a given nonempty set, and the hesitant fuzzy set *H* defined on *T* is a mapping function of a subset from *T* to the interval [0,1].

The aforementioned definition of hesitant fuzzy set can be expressed in mathematical form as follows:

(1) $$\begin{array}{c} H=\left\{t,h_{H}\left(t\right)|t\in T\right\}. \end{array}$$

In formula (1), *h*_*H*_(*t*) is a set of several different real values in the interval [0,1], which means that *t* ∈ *T* represents the possibility of belonging to the hesitant fuzzy set *H* and is the basic element of the hesitant fuzzy set *H*. *h*_*H*_(*t*) is the hesitant fuzzy number and is written as *h*=*h*_*H*_(*t*) for simplicity. The hesitant fuzzy number *h* can be expressed in more detail as *h*=*H*{*γ*^1^, *γ*^2^,…, *γ*^#*h*^}(*γ*^*λ*^ ∈ [0,1],  *λ*=1,2,…, #*h*). Here, #*h* indicates the number of elements in the hesitant fuzzy number *h*. If #*h*=1, that is, the hesitant fuzzy number *h* contains only a single value, then the hesitant fuzzy set *H* degenerates to a traditional fuzzy set, that is, the traditional fuzzy set is a special form of the hesitant fuzzy set, which is called the single-valued hesitant fuzzy set.

As an example, this paper assumes that *T*={bandwidth, delay, jitter, loss, error} is a nonempty set, which represents the set of attributes of a candidate network in an HWN environment. According to the aforementioned definition, it is assumed that the membership degree of *T*={bandwidth, delay, jitter, loss, error} to the hesitant fuzzy set *H* is as follows:

(2) $$\begin{array}{c} \left\{\begin{array}{c} h_{H}\left(\text{bandwidth}\right)=H\left\{0.3,0.1,0.2,0.4,0.5\right\},\\ h_{H}\left(\text{delay}\right)=H\left\{0.2,0.5,0.3,0.4,0.6\right\},\\ h_{H}\left(\text{jitter}\right)=H\left\{0.3,0.6,0.7,0.4,0.5\right\},\\ h_{H}\left(\text{loss}\right)=H\left\{0.5,0.6,0.7,0.4,0.8\right\},\\ h_{H}\left(\text{error}\right)=H\left\{0.5,0.7,0.8,0.6,0.9\right\}. \end{array}\right. \end{array}$$

Formula (2) calls *H* a hesitant fuzzy set of attributes of candidate networks, namely,

(3) $$\begin{array}{c} H=\left\{\text{bandwidth},h_{H}\left(\text{bandwidth}\right)\text{,delay},h_{H}\left(\text{delay}\right)\text{jitter},h_{H}\left(\text{jitter}\right),\text{loss},h_{H}\left(\text{loss}\right)\text{error},h_{H}\left(\text{error}\right)\right\}. \end{array}$$

The hesitant fuzzy number *h*_*H*_(delay)=*H*{0.2, 0.5, 0.3, 0.4, 0.6} in the aforementioned example expressed in the hesitant fuzzy set theory is the membership degree of delay to the hesitant fuzzy set *H* that may be one of 0.2, 0.5, 0.3, 0.4, and 0.6. In the decision-making process of the access selection in this paper, it is assumed that users are hesitant in collecting five specific values of the attribute *de*  *lay* of a candidate network and that these values are possible.

It is apparent that when handling the MADM problem of access selection under hesitation from the hesitant fuzzy number *h*_*H*_(bandwidth), *h*_*H*_(delay), *h*_*H*_(jitter), *h*_*H*_(loss),  and *h*_*H*_(error), the candidate network is permitted to assign multiple possible attribute values, which increases the flexibility of decision-making and can better describe the uncertainty of users on network performance, so as to be more suitable for access-selection decision-making problems in HWNs.

**Step 2 .**

Big-and-small comparison and distance calculation of hesitant fuzzy numbers.

In the MADM process of access selection, the network attribute value of a candidate network is given in the form of the hesitant fuzzy number, that is *h*_*H*_(bandwidth), *h*_*H*_(delay), *h*_*H*_(jitter), *h*_*H*_(loss), and *h*_*H*_(error) discussed in the previous step, the elements in these hesitant fuzzy numbers (namely, possible membership degrees) are usually disordered (i.e., *h*_*H*_(delay)={0.2, 0.5, 0.3, 0.4, 0.6}), and the number of elements in different hesitant fuzzy numbers is usually not equal. It is very difficult to calculate two hesitant fuzzy numbers with disordered elements and an unequal number of the elements, such as when comparing the sizes between them and calculating their distances.

To facilitate calculation, before processing the hesitant fuzzy number, this paper arranges all the elements in the hesitant fuzzy number in ascending order (for example, changing *h*_*H*_(delay)={0.2, 0.5, 0.3, 0.4, 0.6} to *h*_*H*_(delay)={0.2, 0.5, 0.3, 0.4, 0.6}), thus ensuring the order of the elements in the hesitant fuzzy number. In addition, in the access-selection process described herein, it is assumed that the number of elements in the hesitant fuzzy number of each network attribute is 5 (i.e., #*h*=5), which ensures that the number of elements in the hesitant fuzzy number is equal.

Assuming that there are two hesitant fuzzy numbers *h*_1_=*H*{*γ*_1_^*λ*^*|λ*=1,2,…, #*h*} and *h*_2_=*H*{*γ*_2_^*λ*^*|λ*=1,2,…, #*h*}, which have the same number of elements and are arranged in ascending order, this paper makes the following provisions for comparing both hesitant fuzzy numbers:

(4) $$\begin{array}{c} h_{1}\leq h_{2}\text{ if and only if }\gamma_{1}^{\lambda }£\gamma_{2}^{\lambda }\left(\lambda =1,2,L,\#h\right). \end{array}$$

In addition, the distance between two hesitant fuzzy numbers is calculated based on the Euclidean distance as follows:

(5) $$\begin{array}{c} d_{E}\left(h_{1},h_{2}\right)=\sqrt{\frac{1}{\#h}\sum_{\lambda =1}^{\#h}{\left(\gamma_{1}^{\lambda }-\gamma_{2}^{\lambda }\right)}^{2}}. \end{array}$$

**Step 3 .**

Building of a hesitant fuzzy multiattribute matrix.

Let *X*={*x*_1_, *x*_2_,…, *x*_*m*_} be the scheme set composed of four candidate networks, *A*={*a*_1_, *a*_2_,…, *a*_*n*_} be the network attribute set, and the evaluation value *a*_*j*_(*x*_*i*_) of the candidate network *x*_*i*_ (*i*=1,2,…, *m*) under the network attribute *a*_*j*_ (*j*=1,2,…, *n*) be a hesitant fuzzy number, so the decision information matrix ℛ=(*a*_*j*_(*x*_*i*_))_*m*×*n*_ of hesitant fuzzy access selection can be expressed as follows:

(6) $$\begin{array}{c} ℛ=\left[\begin{array}{cccccc} a_{1}\left(x_{1}\right) & a_{2}\left(x_{1}\right) & \cdots & a_{j}\left(x_{1}\right) & \cdots & a_{n}\left(x_{1}\right)\\ a_{1}\left(x_{2}\right) & a_{2}\left(x_{2}\right) & \cdots & a_{j}\left(x_{2}\right) & \cdots & a_{n}\left(x_{2}\right)\\ \vdots & \vdots & \vdots & \vdots & \vdots & \vdots \\ a_{1}\left(x_{i}\right) & a_{2}\left(x_{i}\right) & \cdots & a_{j}\left(x_{i}\right) & \cdots & a_{n}\left(x_{i}\right)\\ \vdots & \vdots & \vdots & \vdots & \vdots & \vdots \\ a_{1}\left(x_{m}\right) & a_{2}\left(x_{m}\right) & \cdots & a_{j}\left(x_{m}\right) & \cdots & a_{n}\left(x_{m}\right) \end{array}\right]. \end{array}$$

In formula (6),

(7) $$\begin{array}{c} a_{j}\left(x_{i}\right)=h_{ij}\\ =H\left\{\gamma_{ij}^{1},\gamma_{ij}^{2},\ldots ,\gamma_{ij}^{\#h}\right\},\;i=1,2,\ldots ,m;\;j=1,2,\ldots ,n. \end{array}$$

In the research scenario of HWNs in this paper, there are four candidate networks, namely UMTS, LTE, WLAN, and WiMAX. The network attributes provided by each candidate network are bandwidth, delay, jitter, packet loss ratio, and BER, and each attribute provides five sets of values when making decisions. Therefore, *m*=4,  *n*=5,  and #*h*=5 in this paper and the attribute values *m*=4,  *n*=5,  and #*h*=5 are expressed by normalized values.

In all network attributes, bandwidth is the benefit attribute (that is, the larger the value, the better the scheme), while delay, jitter, packet loss ratio, and BER are the cost attribute (that is, the smaller the value, the better the scheme). In addition, the numerical range of each network attribute is different (for example, the bandwidth is usually 1 MB/s to 10 MB/s, and the delay is usually 10 ms to 200 ms). To ensure the compatibility among all attributes, this paper normalizes all attribute values and expresses them as values between 0 and 1. Moreover, cost-based attributes are converted into benefit-based attributes. The specific method is as follows:

(8) $$\begin{array}{c} {\bar{h}}_{ij}=\left\{\begin{array}{cc} h_{ij}, & \text{for benefit}-\text{based attributes }a_{j},\\ {\left(h_{ij}\right)}^{c}, & \text{for cost}-\text{based attributes }a_{j}, \end{array}\right. \end{array}$$

where (*h*_*ij*_)^*c*^ is the complementary operation of the hesitant fuzzy number *h*_*ij*_ , that is, (*h*_*ij*_)^*c*^=∪_*γ*Î*h*_*ij*__{1 − *γ*}.

### 3.2. Calculation of Network Attributes Based on the FAHP

The analytic hierarchy process (AHP) is a systematic analysis method combining qualitative and quantitative analysis, which has the advantages of flexibility and conciseness. When there are many evaluation indexes (for example, in this paper, there are five), it is difficult to guarantee the consistency of thinking [31]. Therefore, the FAHP is used to calculate the weights of network attributes. The traditional AHP method establishes the consistent judgment matrix through the pairwise comparison of elements. This paper establishes a fuzzy-consistent matrix using the pairwise comparison of elements [32]. The main calculation steps are as follows:


(9) $$\begin{array}{c} \left\{\begin{array}{c} 0\leq r_{ij}\leq 1,\\ r_{ii}=0.5,\\ r_{ij}=1-r_{ji},\\ r_{ij}=r_{ik}-r_{jk}+0.5, \end{array}\right. \end{array}$$

(10) $$\begin{array}{c} w_{i}=\frac{2}{n\left(n-1\right)}\times \sum_{j=1}^{n}r_{ij}-\frac{1}{n\left(n-1\right)}. \end{array}$$

**Step 4 .**

**Figure 2:**
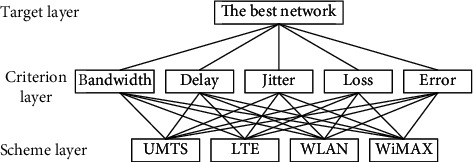
The FAHP's hierarchy.

Analyze the relationship between factors in the access selection of the HWNs and divide the analysis object into the target layer, criteria layer, and scheme layer. Here, the target layer is the best access network, the criterion layer comprises network attributes (i.e., bandwidth, delay, jitter, packet loss ratio, and BER), and the scheme layer is composed of candidate networks (i.e., UMTS, LTE, WLAN, and WiMAX; Figure 2).

**Step 5 .**

Compare attributes in pairs for their importance. By comparing the attribute *x*_*i*_ and the attribute *x*_*j*_, the degree of importance *r*_*ij*_ is obtained. The meaning of the scale of the importance degree is shown (Table 1), and the fuzzy-consistent matrix is built based on *r*_*ij*_. This paper has three different applications (i.e., voice video, and data), and the comparison matrix of importance of attributes under these three applications is shown (Tables 2–4). In addition, the consistency of the matrix can be checked using formula (9), and the weight of each attribute can be calculated according to formula (10).

### 3.3. Calculation of Candidate Network Scores Using the Hesitant Fuzzy TOPSIS Method

The TOPSIS algorithm mainly evaluates the candidate network's closeness to the best network and the worst network. The most ideal situation is that the evaluated network is closest to the best network and is farthest from the worst network [33]. This paper proposes a hesitant fuzzy TOPSIS method to score and rank candidate networks based on the traditional TOPSIS method. The specific calculation steps are as follows:

**Step 6 .**

Determine the hesitant fuzzy-positive ideal solution (PIS) *x*^+^ and the hesitant fuzzy-negative ideal solution (NIS) *x*^−^.

Generally speaking, the hesitant fuzzy PIS *x*^+^ and NIS *x*^−^ are usually not one of the candidate schemes, that is, *x*^+^, *x*^−^ ∉ *X*; otherwise, *x*^+^ is the best among all candidate schemes. In addition, all their attribute values are equal to those of any candidate scheme, while all their attribute values of *x*^−^ are inferior to those of any candidate scheme. Therefore, formulas (11) and (12) are used to determine the hesitant fuzzy PIS *x*^+^ and NIS *x*^−^, respectively.

(11) $$\begin{array}{c} x^{+}=\left\{a_{j},\underset{i=1,2,\ldots ,m}{\max}\gamma_{ij}^{\lambda }|j=1,2,\ldots ,n,\;\lambda =1,2,\ldots ,\#h\right\}\\ =\left\{\langle a_{1}H\left\{{\left(\gamma_{1}^{1}\right)}^{+},{\left(\gamma_{1}^{2}\right)}^{+},\ldots ,{\left(\gamma_{1}^{\#h}\right)}^{+}\right\}\rangle ,\langle a_{2},H\left\{{\left(\gamma_{2}^{1}\right)}^{+},{\left(\gamma_{2}^{2}\right)}^{+},\ldots ,{\left(\gamma_{2}^{\#h}\right)}^{+}\right\}\rangle \ldots ,\langle a_{n},H\left\{{\left(\gamma_{n}^{1}\right)}^{+},{\left(\gamma_{n}^{2}\right)}^{+},\ldots ,{\left(\gamma_{n}^{\#h}\right)}^{+}\right\}\rangle \right\}, \end{array}$$

(12) $$\begin{array}{c} x^{-}=\left\{a_{j},\underset{i=1,2,\ldots ,m}{\max}\gamma_{ij}^{\lambda }|j=1,2,\ldots ,n,\;\lambda =1,2,\ldots ,\#h\right\}\\ =\left\{\langle a_{1}H\left\{{\left(\gamma_{1}^{1}\right)}^{-},{\left(\gamma_{1}^{2}\right)}^{-},\ldots ,{\left(\gamma_{1}^{\#h}\right)}^{-}\right\}\rangle ,\langle a_{2},H\left\{{\left(\gamma_{2}^{1}\right)}^{-},{\left(\gamma_{2}^{2}\right)}^{-},\ldots ,{\left(\gamma_{2}^{\#h}\right)}^{-}\right\}\rangle \ldots ,\langle a_{n},H\left\{{\left(\gamma_{n}^{1}\right)}^{-},{\left(\gamma_{n}^{2}\right)}^{-},\ldots ,{\left(\gamma_{n}^{\#h}\right)}^{-}\right\}\rangle \right\}, \end{array}$$

**Step 7 .**

Calculate the distance between the candidate network *x*_*i*_ and the hesitant fuzzy PIS *x*^+^ and NIS *x*^−^.

In Step 6, the PIS *x*^+^ and the NIS *x*^−^ are obtained. According to the calculation method of the fuzzy Euclidean distance in formula (5), the distance between each candidate network *x*_*i*_ and the fuzzy-hesitant PIS *x*^+^ and NIS *x*^−^ is determined using formulas (13) and (14) in Step 7, which are respectively written as follows:

(13) $$\begin{array}{c} D\left(x_{i},x^{+}\right)=\sum_{j=1}^{n}d_{E}\left(h_{ij},h_{j}^{+}\right)w_{j}\\ =\sum_{j=1}^{n}w_{j}\sqrt{\frac{1}{\#h}\sum_{\lambda =1}^{\#h}{\left(\gamma_{ij}^{\lambda }-{\left(\gamma_{j}^{\lambda }\right)}^{+}\right)}^{2}}, \end{array}$$

(14) $$\begin{array}{c} D\left(x_{i},x^{-}\right)=\sum_{j=1}^{n}d_{E}\left(h_{ij},h_{j}^{-}\right)w_{j}\\ =\sum_{j=1}^{n}w_{j}\sqrt{\frac{1}{\#h}\sum_{\lambda =1}^{\#h}{\left(\gamma_{ij}^{\lambda }-{\left(\gamma_{j}^{\lambda }\right)}^{-}\right)}^{2}}. \end{array}$$

The smaller *D*(*x*_*i*_, *x*^+^) is, the closer the distance between *x*_*i*_ and *x*^+^, which means the better *x*_*i*_ is. In addition, the greater *D*(*x*_*i*_, *x*^−^) is, the farther the distance between *x*_*i*_ and *x*^−^ , and the better *x*_*i*_ is. Therefore, use *D*_min_(*x*_*i*_, *x*^+^) to represent the distance from the nearest candidate network *x*^+^ and *D*_max_(*x*_*i*_, *x*^−^) to represent the distance from the farthest candidate network *x*^−^:

(15) $$\begin{array}{c} D_{\text{min}}\left(x_{i},x^{+}\right)=\underset{1\leq i\leq m}{\min}D\left(x_{i},x^{+}\right),\\ D_{\text{max}}\left(x_{i},x^{-}\right)=\underset{1\leq i\leq m}{\max}D\left(x_{i},x^{-}\right). \end{array}$$

**Step 8 .**

Calculate the relative closeness between the candidate network *x*_*i*_ and the hesitant fuzzy PIS *x*^+^.

(16) $$\begin{array}{c} \text{Score}\left(x_{i}\right)=\frac{D\left(x_{i},x^{-}\right)}{D\left(x_{i},x^{-}\right)+D\left(x_{i},x^{+}\right)}, \end{array}$$

Score(*x*_*i*_) in formula (16) represents the final score of candidate network *x*_*i*_. It is apparent that the greater the value of 0 ≤ Score(*x*_*i*_) ≤ 1 (*i*=1,2,…, *m*), Score(*x*_*i*_), the better the candidate network *x*_*i*_ is.

## 4. Simulation and Results Analysis

### 4.1. Experimental Environment and Simulation Parameter Settings

This paper uses MATLAB R2019b as the simulation platform to test and compare the algorithms mentioned herein. In the simulation experiment, the network attribute values of each candidate network are set (Table 5), with the values in brackets indicating the lowest value and the highest value of the network attribute when it changes dynamically.

To prove the superiority of the algorithm proposed in this paper, this algorithm is compared with another three algorithms proposed in other literature (i.e., the AHP and TOPSIS algorithms by Habbal et al. [23]; the Utility, TFAHP, and TOPSIS algorithms by Goyal et al. [24]; and the fuzzy logic algorithm by Ahuja et al. [25], which are called Algorithm 1, Algorithm 2, and Algorithm 3, respectively). For fairness, the weights of network attributes for the algorithm proposed herein under each application are set to be the same as the other three algorithms.

The experiment mainly consists of two parts. The first part is to select a network using the proposed algorithm under the dynamic network attribute environment, which mainly evaluates the average value of the network attributes using the proposed algorithm under various applications and the number of selections of each candidate network. The second part is to compare the performance of these algorithms, mainly comparing the number of selections of candidate networks, number of handoffs between networks, number of unnecessary handoffs, and average user gain under different applications.

### 4.2. Network Selection under Dynamic Network Attribute Environment Using the Proposed Algorithm

**Figure 3:**
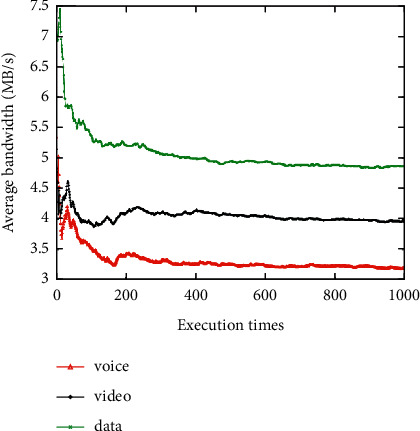
Average bandwidth value of the selected network.

**Figure 4:**
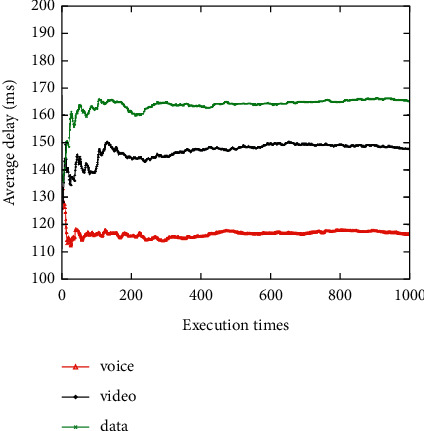
Average delay value of the selected network.

**Figure 5:**
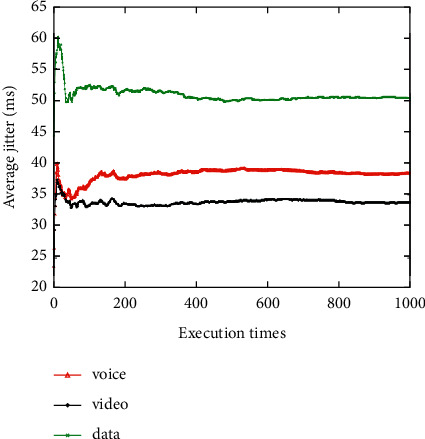
Average jitter value of the selected network.

**Figure 6:**
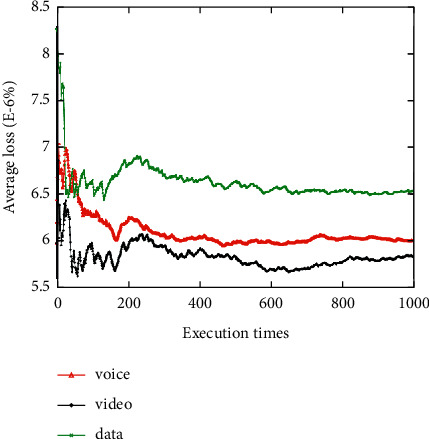
Average packet loss ratio value of the selected network.

**Figure 7:**
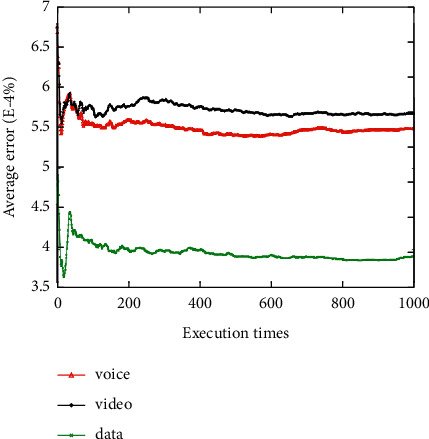
Average bit error ratio value of the selected network.

The average network attribute values of the networks selected for the voice, video, and data applications are shown when the network attribute value changes dynamically 1000 times (Figures 3–7). It can be seen that among the networks selected by the three applications, the voice application only needs lower bandwidth to meet its service requirements, and the weight of bandwidth under the voice application is the lowest (Figure 3). Therefore, the network selected by the voice application has the lowest average bandwidth value in 1000 network selections. On the contrary, as the data application has a higher bandwidth demand, the average bandwidth value of the selected network in the data application is the highest. It can be seen that among the three applications, the network selected for the voice application has the lowest average delay value, while the network selected for the video application has the lowest average jitter value (Figures 4 and 5). The reason is that the voice application is sensitive to delay, while the video application has higher requirements for jitter. It can be seen that the average packet loss ratio of the network selected for the video application is the lowest because the weight of packet loss ratio is larger in the video application (Figure 6). It can also be seen that the data application has high requirements for BER, so the average BER of the network selected for the data application is the lowest (Figure 7). In general, it appears that the algorithm proposed in this paper can select the most suitable network for users according to the characteristics of each application and the weights of different network attributes for different applications (Figures 3–7).

**Figure 8:**
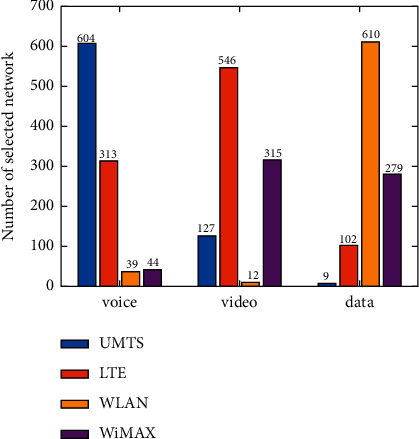
Number of candidate network selection under different applications.

The number of selections of each candidate network is shown under different applications (Figure 8). As the voice application does not need high bandwidth but still requires a low delay guarantee, UMTS is the most selected network for the voice application, followed by LTE. Meanwhile, WLAN and WiMAX are less frequently selected. The video application needs a low jitter and packet loss ratio, as well as a certain bandwidth guarantee. Therefore, LTE is the most selected network for the video application, while WiMAX is also selected for a certain number of times. For the data application, a higher bandwidth guarantee is required, so WLAN is the most selected network for the data application, while UMTS is the least selected network. It can be seen that the algorithm proposed herein can select the most suitable network according to the service characteristics under the environment of dynamically changing network attributes (Figure 8).

### 4.3. Comparison of Algorithms

This section compares the algorithm proposed in this paper with the other three algorithms. Moreover, it analyzes the number of selections of the network, the number of handoffs between the networks, the number of unnecessary handoffs, and user gain.

**Figure 9:**
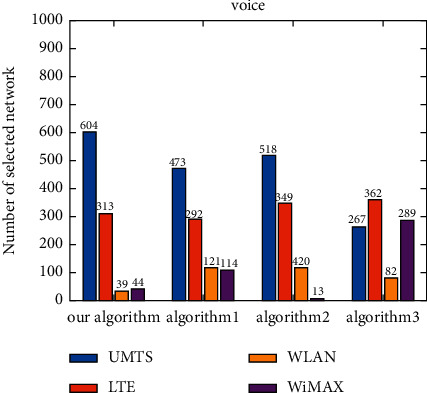
Number of candidate network selection for voice.

**Figure 10:**
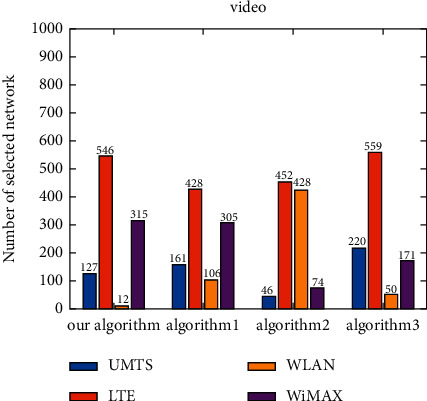
Number of candidate network selection for video.

**Figure 11:**
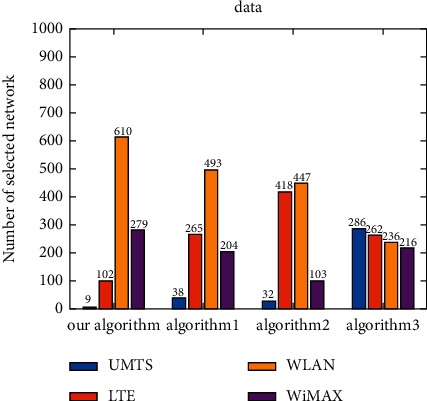
Number of candidate network selection for data.

Under the voice application, UMTS is the most frequently selected network by the proposed algorithm, Algorithm 1, and Algorithm 2, followed by LTE, and these three algorithms choose WLAN and WiMAX less frequently (Figure 9). The network most frequently selected by Algorithm 3 is LTE, and UMTS and WiMAX are also selected for a certain number of times. Under the video application, the network which is most selected by all algorithms is LTE (Figure 10). WiMAX is selected for a certain number of times by the proposed algorithm and Algorithm 1. Algorithm 2 chooses WLAN for a similar number of times as LTE. In addition, Algorithm 3 chooses UMTS for a certain number of times. Under the data application, WLAN is the most frequently selected network by the proposed algorithm, Algorithm 1, and Algorithm 2, and the network most frequently selected by Algorithm 3 is UMTS (Figure 11). For the proposed algorithm, the number of selections of four candidate networks is obviously different, while for Algorithm 3, the number of selections of four candidate networks is very close.

**Figure 12:**
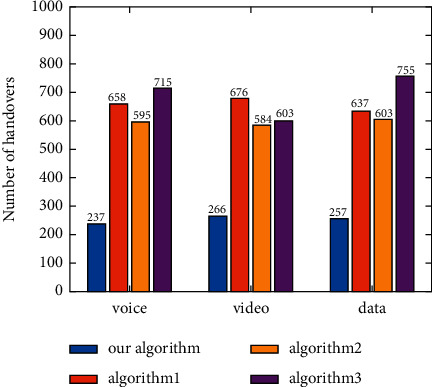
Number of handoffs with different applications.

The number of handoffs between networks under different applications is compared (Figure 12). Under the voice application, the proposed algorithm mainly selects UMTS and LTE (Figure 9), and the handoffs mainly occur between UMTS and LTE, so that the number of handoffs is only 237. As the number of selections of UMTS, LTE, and WiMAX by Algorithm 3 is relatively close, there are more than 700 handoffs. Under the video application, the number of selections of the four candidate networks made by Algorithm 1 exceeds 100, so Algorithm 1 has a higher number of handoffs than other algorithms. As the proposed algorithm mainly selects LTE and WiMAX, and WLAN is only selected for 12 times (Figure 10), the proposed algorithm only has 266 handoffs. Under the data application, as the number of selections of four candidate networks made by Algorithm 3 is very close (Figure 11), there are more than 750 handoffs. The handoffs caused by the proposed algorithm mainly occur between WLAN and WiMAX, which is 257. In general, the number of handoffs caused by the proposed algorithm is lower than those caused by the other three algorithms under different applications.

**Figure 13:**
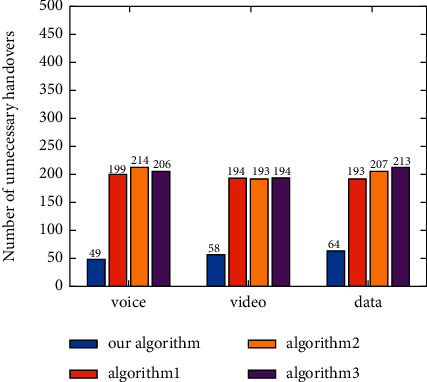
Number of unnecessary handoffs with different applications.

According to the definition of “Unnecessary Handoff” given by Yu et al. [34], the number of unnecessary handoffs caused by each algorithm under different applications is counted. Under the applications of voice, video, and data, the number of unnecessary handoffs between different networks caused by the proposed algorithm is about 50 and 200 caused by other algorithms (Figure 13).

Index gain is a measure of user satisfaction in the access-selection process. According to Yu and Zhang [26], the definition of gain for this paper is shown in formula (17):

(17) $$\begin{array}{c} G_{i}=\lambda \prod_{k=1}^{n}r_{k}^{\omega_{k}}, \end{array}$$

where *G*_*i*_ represents the gain achieved by users in the network *i*, *n* represents the number of network attributes (in this paper, *n*=5), *r*_*k*_ represents the normalized value of network attribute *k*, and *ω*_*k*_ represents the weight of network attribute *k*. In addition, for *λ* in formula (17), when a user selects the same network twice consecutively, let *λ*=1, and when the user selects different networks twice consecutively, let *λ*=0.8.

**Figure 14:**
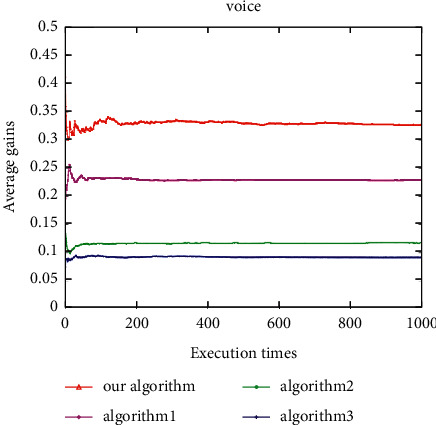
Average user gain for voice.

**Figure 15:**
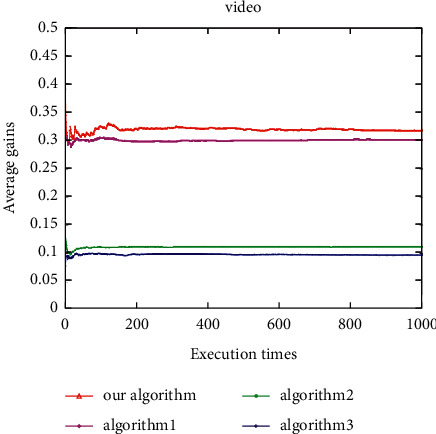
Average user gain for video.

**Figure 16:**
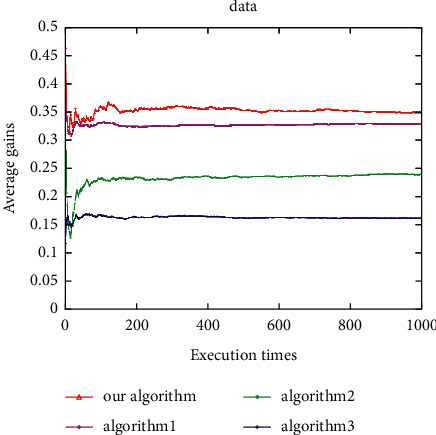
Average user gain for data.

Under the voice application, the average user gain of this algorithm is better than Algorithm 1, and both are better than Algorithm 2 and Algorithm 3 (Figure 14). The proposed algorithm is better than all other algorithms under the video application (Figure 15). In addition, under the data application, the average gain of the proposed algorithm is the highest, followed by Algorithm 1, Algorithm 2, and Algorithm 3 (Figure 16). It can be seen that the average gain of the proposed algorithm under different applications is higher than that of the other three algorithms (Figures 14–16). This is due to the proposed algorithm being able to select the most suitable network for users according to uncertain network attribute values as the environment has constantly fluctuating network attribute values. In addition, as other algorithms can cause user terminals to frequently switch between different networks, which cannot reduce the ping-pong effect, the average gain is low, and the algorithm proposed herein can reduce the number of handoffs between different networks and ensure that the users have a better quality of experience (QoE).

## 5. Conclusion

In an HWN environment, the network attribute values obtained by users are often uncertain due to the mobility of users, the interference of wireless signals, and the fluctuation of the network state. To solve this problem, this paper designs an access-selection algorithm for HWNs in the context of inaccurate network attribute values, which integrates the hesitant fuzzy theory, the FAHP, and the MADM. First, the algorithm uses the hesitant fuzzy theory to calculate network attribute values, then uses the FAHP to calculate the weights of network attributes, and finally uses the hesitant fuzzy TOPSIS method to sort candidate networks. The simulation results show that the algorithm proposed in this paper enables users to access the most suitable network under the environment of inaccurate network attribute values. Consequently, it reduces the number of handoffs between different networks and enables users to obtain higher gains.

The framework of this algorithm can be applied to various decision-making scenarios, such as construction contractor selection, destination selection, and benefit evaluation [35]. Future research work will further consider factors such as the interval of inaccurate network attribute values and the characteristics of different applications to obtain better QoS support and user experience.

## Figures and Tables

**Table 1:** Interpretation for importance level.

Level	Interpretation
0.5	Both are equally important
0.6	The former is slightly more important than the latter
0.7	The former is obviously more important than the latter
0.8	The former is strongly more important than the latter
0.9	The former is extremely more important than the latter
0.1, 0.2, 0.3, and 0.4	If *x*_*i*_ is compared with *x*_*j*_, then *r*_*ij*_ is obtained. *x*_*j*_ and *x*_*i*_ can be compared to the results in *r*_*ji*_=1 − *r*_*ij*_. 0.55, 0.65, and 0.75, and the like represent the median value of adjacent levels.

**Table 2:** Fuzzy-consistent matrix and weights for voice application.

Voice	Bandwidth	Delay	Jitter	Loss	Error	Weight
Bandwidth	0.5	0.15	0.3	0.45	0.35	0.1250
Delay	0.85	0.5	0.65	0.8	0.7	0.3000
Jitter	0.7	0.35	0.5	0.65	0.55	0.2250
Loss	0.55	0.2	0.35	0.5	0.4	0.1500
Error	0.65	0.3	0.45	0.6	0.5	0.2000

**Table 3:** Fuzzy-consistent matrix and weights for video application.

Video	Bandwidth	Delay	Jitter	Loss	Error	Weight
Bandwidth	0.5	0.65	0.25	0.4	0.7	0.2000
Delay	0.35	0.5	0.1	0.25	0.55	0.1250
Jitter	0.75	0.9	0.5	0.65	0.95	0.3250
Loss	0.6	0.75	0.35	0.5	0.8	0.2500
Error	0.3	0.45	0.05	0.2	0.5	0.1000

**Table 4:** Fuzzy-consistent matrix and weights for data application.

Data	Bandwidth	Delay	Jitter	Loss	Error	Weight
Bandwidth	0.5	0.95	0.85	0.7	0.6	0.3100
Delay	0.05	0.5	0.4	0.25	0.15	0.0850
Jitter	0.15	0.6	0.5	0.35	0.25	0.1350
Loss	0.3	0.75	0.65	0.5	0.4	0.2100
Error	0.4	0.85	0.75	0.6	0.5	0.2600

**Table 5:** Network attribute values of candidate networks.

	Bandwidth (MB/s)	Delay (ms)	Jitter (ms)	Loss (*E*-6%)	Error (*E*-4%)
UMTS	0.5–2	20–150	20–50	2–8	3–7
LTE	0.8–8	30–200	10–30	3–10	4–8
WLAN	1–10	50–250	30–80	4–12	1–5
WiMAX	0.6–4	80–300	15–40	1–5	2–6

## Data Availability

MATLAB code and experimental data can be downloaded from the following link (link: https://pan.baidu.com/s/18X7o0-AMQBXr6fimYUZf8g; password: rwbk; password: x0pb).
